# Structure of the 4-O-[1-Carboxyethyl]-d-Mannose-Containing O-Specific Polysaccharide of a Halophilic Bacterium *Salinivibrio* sp. EG9S8QL

**DOI:** 10.3390/md19090508

**Published:** 2021-09-07

**Authors:** Elena N. Sigida, Ibrahim M. Ibrahim, Maxim S. Kokoulin, Hussein H. Abulreesh, Khaled Elbanna, Svetlana A. Konnova, Yulia P. Fedonenko

**Affiliations:** 1 Institute of Biochemistry and Physiology of Plants and Microorganisms, Russian Academy of Sciences, 13 Prospekt Entuziastov, 410049 Saratov, Russia; konnovasa@yandex.ru (S.A.K.); fedonenko_yu@ibppm.ru (Y.P.F.); 2 N. D. Zelinsky Institute of Organic Chemistry, Russian Academy of Sciences, 47 Leninsky Prospekt, 119991 Moscow, Russia; 3 N. G. Chernyshevsky Saratov State University, 83 Ulitsa Astrakhanskaya, 410012 Saratov, Russia; imb00@fayoum.edu.eg; 4 Department of Agricultural Microbiology, Faculty of Agriculture, Fayoum University, Fayoum 63514, Egypt; kab00@fayoum.edu.eg; 5 G. B. Elyakov Pacific Institute of Bioorganic Chemistry, Far Eastern Branch of Russian Academy of Sciences, 159 Prospekt 100 let Vladivostoku, 690022 Vladivostok, Russia; maxchem@mail.ru; 6 Department of Biology, Faculty of Applied Science, Umm Al-Qura University, Makkah 21955, Saudi Arabia; hhabulreesh@uqu.edu.sa; 7 Research Laboratories Unit, Faculty of Applied Science, Umm Al-Qura University, Makkah 21955, Saudi Arabia

**Keywords:** lipopolysaccharide, O-specific polysaccharide, structure, *Salinivibrio*, halophilic bacterium, 4-O-[1-carboxyethyl]-d-mannose

## Abstract

The moderately halophilic strain *Salinivibrio* sp. EG9S8QL was isolated among 11 halophilic strains from saline mud (Emisal Salt Company, Lake Qarun, Fayoum, Egypt). The lipopolysaccharide was extracted from dried cells of *Salinivibrio* sp. EG9S8QL by the phenol–water procedure. The OPS was obtained by mild acid hydrolysis of the lipopolysaccharide and was studied by sugar analysis along with ^1^H and ^13^C NMR spectroscopy, including ^1^H,^1^H COSY, TOCSY, ROESY, ^1^H,^13^C HSQC, and HMBC experiments. The OPS was found to be composed of linear tetrasaccharide repeating units of the following structure: →2)-β-Man*p*4Lac-(1→3)-α-Man*p*NAc-(1→3)-β-Rha*p*-(1→4)-α-Glc*p*NAc-(1→, where Man*p*4Lac is 4-O-[1-carboxyethyl]mannose.

## 1. Introduction

Microorganisms adapted to extreme environmental conditions, unsuitable for the survival of other organisms, are called extremophiles. They include archaea, halophilic and halotolerant bacteria, and algae. Among other factors, salinity strongly affects the composition of the microbial communities of water areas [1,2]. Saline environments are found everywhere on Earth and include seas, thermal springs, salterns, and lakes. In endorheic lakes, which are not connected to the world ocean by river systems, water is lost through evaporation or percolation. As a result, minerals and other erosion products accumulate. Endorheic lakes are used widely to produce mineral salts. The unique hydrological regime, seasonal changes in salinity, and human activities greatly affect microbial diversity in such lakes [3,4].

Moderately halophilic Gram-negative gammaproteobacteria of the genus *Salinivibrio* within the family Vibrionaceae are normal inhabitants of hypersaline systems including marine environments worldwide [5,6]. They have been isolated from saline lakes, soils, salterns, and fermented food [5,6,7,8,9,10]. The genus *Salinivibrio* comprises six species, including *S. costicola*, *S. kushneri*, *S. proteolyticus*, *S. sharmensis*, *S. siamensis*, and *S. socompensis* [11]. *Salinivibrio* bacteria are tolerant to various biotic and abiotic stress factors, such as osmotic shock and exposure to alkalis, heavy metals, and other toxic compounds [12]. These bacteria are of high biotechnological interest, because they can degrade highly toxic dyes, including diazo dyes [13], and produce polyhydroxyalcanoates [14] and ectoines [15]. Research on the characteristics determining the high adaptation potential of *Salinivibrio* bacteria revealed redundancy of their genome [12].

The type of strain of *S. costicola*, capable of growing over a wide range of salt concentrations, is a model object for studies of the physiological mechanisms of haloadaptation and osmoregulation [16]. To resist osmotic stress, halophilic bacteria use two alternative strategies—“salt-in” and “salt-out” [17]. The “salt-in” strategy is the accumulation of salt in the bacterial cell, whereas the “salt-out” strategy is the exclusion of salt from the cell and the subsequent immediate accumulation of osmolytes, which protect the cell from low water conditions. Both strategies involve the bacterial cell envelope, which enables cell communication with the environment [16,17]. This is why bacterial cell wall components are thought to be key elements of the adaptation mechanisms of halophiles.

The major component of the outer leaflet of Gram-negative bacteria is lipopolysaccharide (LPS), a molecule essential for bacterial viability. LPS contributes to the outer membrane permeability barrier and is essential for membrane integrity. Chemically, LPS is a glycolipid composed of three portions covalently linked to each other: lipid A, embedded in the membrane; a core oligosaccharide; and an O-polysaccharide (OPS), projecting into the cell surroundings [18]. So far, the structures of the core oligosaccharide and lipid A of *S. sharmensis* BAG^T^ have been reported [19,20], but there have been no data on the OPS structure in any *Salinivibrio* sp. [21].

Herein, we report on the chemical structure of the OPS from *Salinivibrio* sp. EG9S8QL isolated from Lake Qarun (Egypt).

## 2. Results

### 2.1. Strain Isolation and Identification

The strain *Salinivibrio* sp. EG9S8QL was isolated from among 11 halophilic strains from saline mud (Emisal Salt Company, Lake Qarun, Fayoum, Egypt). Bacteria formed round, cream-white, creamy colonies on a solid LB nutrient medium supplemented with 5% NaCl. Strain EG9S8QL can utilize a moderate range of carbon sources, including casein, gelatin, tween 80 (Supplementary Material, Table S1). The optimum growth conditions were 10% (*w*Egypt*v*) NaCl, pH 8.0, 30 °C, and sucrose as a carbon source. Phylogenetic analysis of 16S rDNA placed strain EG9S8QL firmly into the genus *Salinivibrio.* Strain EG9S8QL was clustered with *S. kushneri* strain AL184 (NR157685.1) (99.78% identity), *S. costicola* strain Marseille-P2423 (LT223681.1) (99.78% identity) and *S. costicola* subsp. costicola strain CECT 4059 (LT722673.1) (99.12% identity) (Supplementary Material, Figure S1). The phylogenetic affinity of the 16S rDNA gene sequence of *Salinivibrio* spp. and some phenotypic differences between them (Supplementary Material, Table S1) do not allow us to unambiguously assign strain EG9S8QL to any known species of this genus.

### 2.2. LPS Isolation and Characterization 

The LPS was isolated from the cells *Salinivibrio* sp. EG9S8QL by hot phenol–water extraction [22]. The fatty acid composition of the LPS obtained by GC analysis of their methyl ester derivatives showed the prevalence of 3-hydroxydodecanoic acid [12:0(3-OH)] (~36%) and 3-hydroxytetradecanoic acid [14:0(3-OH)] (~33%), as well as dodecanoic acid [12:0] (~13%), tetradecanoic acid [14:0] (~5%) and hexadecanoic acid [16:0] (~13%). O-Deacylation of the LPS with NH_4_OH revealed that 12:0(3-OH) is a primary O-linked fatty acid. Taking into account the conservativeness of the lipid A structure within each bacterial genus, we can suggest that the lipid A of *Salinivibrio* sp. EG9S8QL is structurally close to that of *S. sharmensis* BAG^T^ [20]. SDS-PAGE of the LPS demonstrated the presence of molecules containing the OPS, as well as intensively stained fast-migrating fractions corresponding to the R-form (Supplementary Material, Figure S2). The electrophoretic profile of the LPS was typical for molecules with relatively short O-chains.

### 2.3. Structural Analysis of the OPS

**Figure 1:**
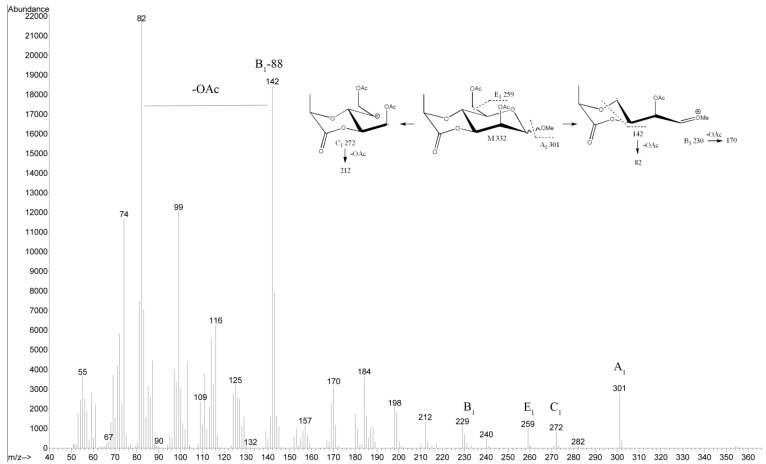
EI mass spectrum of the acetylated methyl glycoside of 4-O-[1-carboxyethyl]-d-mannose in a lactone form.

The LPS of *Salinivibrio* sp. EG9S8QL was obtained from dried cells by the phenol–water extraction and was degraded under mild acidic conditions. After the removal of lipid A by centrifugation, the OPS-containing supernatant was fractionated by gel-permeation-chromatography on a Sephadex G-50 F column. The yield of the OPS fraction was 29% of the LPS mass. The GLC sugar analysis of alditol acetates obtained after hydrolysis of the OPS in 2 M TFA showed the presence of rhamnose (Rha), mannosamine (ManN) and glucosamine (GlcN) as the major components, in a peak area ratio of 1.0:0.5:0.6. Additionally, the GC–MS analysis of the acetylated methyl glycosides showed the presence of a hexose carrying 2-hydroxypropanoyl residue, identified later by NMR as 4-O-[1-carboxyethyl]-d-mannose (see below). The electron impact EI mass spectrum of the latter compound in a lactone form as an acetylated methyl glycoside showed characteristic ions at *m*/*z* 301, 272, 259, 230, and 142 (Figure 1). GC of the acetylated (*S*)-2-octyl glycosides revealed the l absolute configuration of Rha and the d configuration of other monosaccharides.

**Figure 2:**
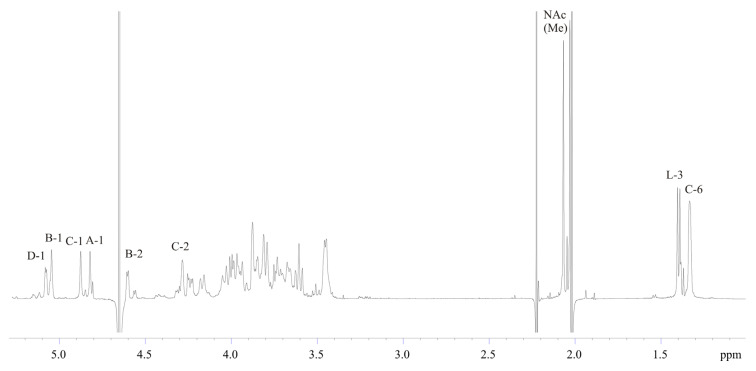
^1^H NMR spectrum of the OPS from *Salinivibrio* sp. EG9S8QL. Arabic numerals refer to protons and carbons in sugar residues denoted by letters as indicated in Table 1.

The structure of the OPS was examined by NMR spectroscopy. The major series of ^1^H NMR spectrum of the OPS (Figure 2) contained signals for four anomeric protons at δ 4.82–5.07, two *C*H_3_-C groups (H-6 of Rha) at δ 1.33 and H-3 of lactic acid (Lac) at δ 1.40, signals of *N*-acetyl groups at δ 2.07, and other sugar protons at δ 3.45–4.60. Along with the major series in the ^1^H NMR spectrum, a minor series of signals was observed, which presumably originated from the core oligosaccharide attached to the O-chain.

**Figure 3:**
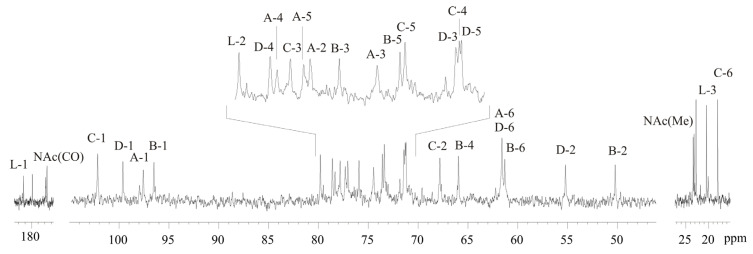
^13^C NMR spectrum of the OPS from *Salinivibrio* sp. EG9S8QL. Arabic numerals refer to protons and carbons in sugar residues denoted by letters as indicated in Table 1.

Accordingly, the ^13^C NMR spectrum of the OPS (Figure 3) contained signals for four carbons in the anomeric region at δ 96.5–102.2, three HO*C*H_2_-C groups (C-6 of D-GlcN, D-ManN and D-Man4Lac) at δ 61.3 and 61.6 (double intensity), two nitrogen-bearing carbons at δ 50.2 and 55.2 (C-2 of D-GlcN and D-ManN), two *C*H_3_-C groups (C-6 of L-Rha) at δ 18.0 and C-3 of Lac at δ 20.5, two signals of NAc-groups at δ 23.5–23.7 (CH_3_) and 177.7–177.8 (CO), as well as fifteen HO*C*H-C groups, including C-2 of Lac, in the region of δ 65.9–79.8. Therefore, the OPS consists of tetrasaccharide repeating units and contains O-linked Lac residue (characteristic signals at δ_C_ 182.5, 79.8, and 20.5). The absence of signals in the region δ 83–88 characteristic for furanosides revealed that all monosaccharides in the OPS are in pyranose form.

**Figure 4:**
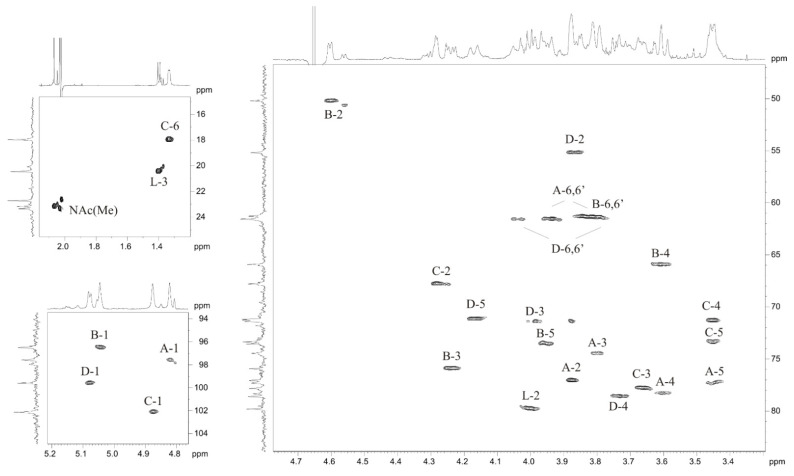
^1^H,^13^C HSQC spectrum of the OPS from *Salinivibrio* sp. EG9S8QL. Arabic numerals refer to protons and carbons in sugar residues denoted by letters as indicated in Table 1.

The ^1^H and ^13^C NMR spectra of the OPS were assigned using ^1^H,^1^H COSY, TOCSY, ROESY, ^1^H,^13^C HSQC (Figure 4), and HMBC experiments (Table 1). Based on ^13^C NMR chemical shifts, intraresidue ^1^H,^1^H and ^1^H,^13^C correlations and ^3^*J*_H,H_ coupling constant values, spin systems of Lac and four monosaccharide residues were identified, including three *manno*-configurated sugars, D-Man*p*4Lac, D-Man*p*NAc and L-Rha*p* (units **A**, **B** and **C**), and one *gluco*-configurated sugar, D-Glc*p*NAc (unit **D**). The TOCSY spectrum showed H-1/H-2 and H-2/H-3,4,5,6 cross-peaks for units **A**–**C** and H-1/H-2,3,4,5,6 cross-peaks for unit **D**. The signals within each spin system were assigned using the ^1^H,^1^H-COSY spectrum.

The presence of Lac at position 4 of residue **A** was confirmed on the basis of a strong correlations between **A** H-4 and C-2 of Lac at δ 3.60/79.8 and vice versa, H-2 of Lac and **A** C-4 at δ 4.00/78.3 in the ^1^H,^13^C HMBC spectrum. 

The α configuration of units **B** and **D** and the β configuration of units **A** and **C** were inferred from characteristic chemical shifts of the C-5 signals as compared with published data of the corresponding α- and β-methyl glycosides [23].

**Figure 5:**
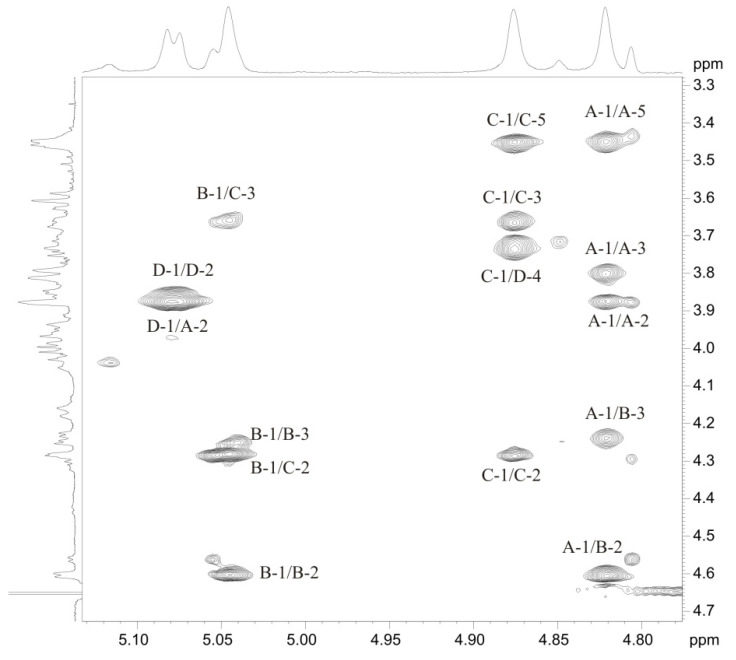
Sections of ^1^H,^1^H ROESY spectrum of the OPS from *Salinivibrio* sp. EG9S8QL. Arabic numerals before and after slash refer to protons in sugar residues denoted by letters as indicated in Table 1.

**Figure 6:**
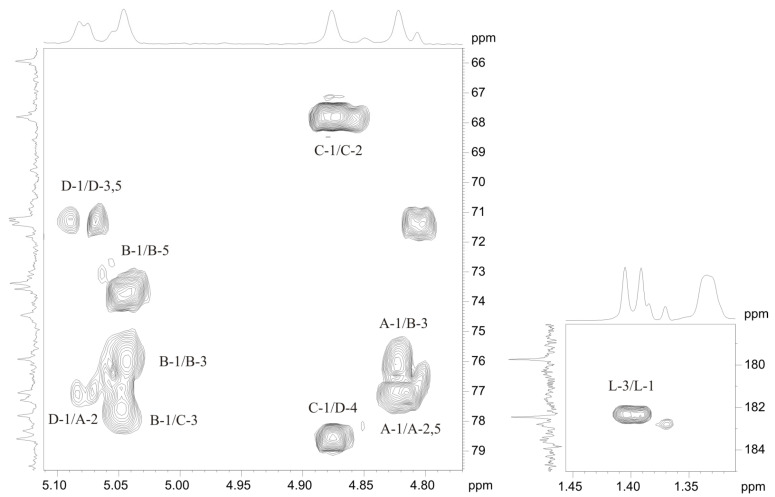
Sections of ^1^H,^13^C HMBC spectrum of the OPS from *Salinivibrio* sp. EG9S8QL. Arabic numerals before and after slash refer to protons and carbons, respectively, in sugar residues denoted by letters as indicated in Table 1.

The linkage analysis of sugar residues in the OPS was performed using ^1^H,^1^H-ROESY and ^1^H,^13^C-HMBC experiments. The following interresidue correlations between anomeric protons and protons at the linkage carbons were observed in the ROESY spectrum of the OPS: **A** H-1/**B** H-3 at δ 4.82/4.24; **B** H-1/**C** H-3 at δ 5.05/3.67, **C** H-1/**D** H-4 at δ 4.88/3.74, **D** H-1/**A** H-2 at δ 5.07/3.88 (Figure 5). The ^1^H,^13^C HMBC experiment (Figure 6) demonstrated the respective correlations between the following anomeric protons and transglycosidic carbons: **A** H-1/**B** C-3 at δ 4.82/75.9; **B** H-1/**C** C-3 at δ 5.05/77.8, **C** H-1/**D** C-4 at δ 4.88/78.6, **D** H-1/**A** C-2 at δ 5.07/77.1. Significant downfield displacements of the C-3 signals of residues **B** and **C**, C-2 signal of residue **A**, and C-4 signals of residue **D**, as compared with their positions in non-substituted monosaccharides [23,24], confirmed the glycosylation pattern and revealed the monosaccharide sequence in the repeating unit.

**Figure 7:**
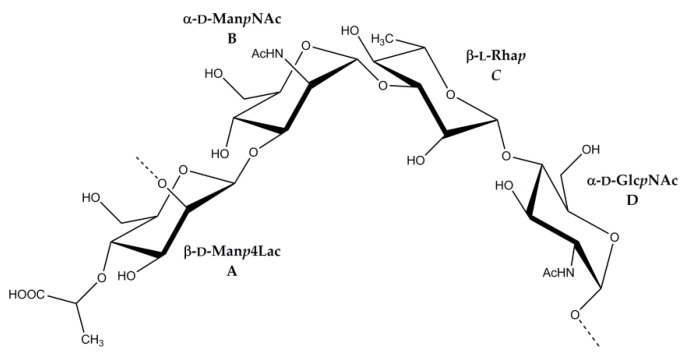
Structure of the OPS from *Salinivibrio* sp. EG9S8QL.

Therefore, the OPS consists of linear tetrasaccharide repeating units (Figure 7): 

## 3. Discussion

Living in the extreme conditions of saline lakes, which are characterized by a higher salt concentration than seawater, a high level of ultraviolet radiation and heavy metals, has led to a diversification of adaptive mechanisms in halophilic microorganisms. These include production of polysaccharides and other biopolymers and/or modification of their structure. A high level of extracellular polysaccharide production increases water retention and heavy metals binding [16]. The ratio between proteins and polysaccharides of the bacterial surface and their structural features is essential for physicochemical characteristics of cell surface such as hydrophobicity, surface charge, etc., important for the adhesion of bacteria to various surfaces, intercellular aggregation and biofilm formation [25,26]. The predominant component of the outer membrane of Gram-negative bacteria, lipopolysaccharide, makes a significant contribution to the formation of a negative charge on the bacterial cell surface, facilitating the electrostatic binding of polyvalent cations and promoting cell aggregation [27].

Glycans of extremophiles are often unique in structure and properties [28] but their structural and functional roles, including in the process of haloadaptation, have not been studied enough. Studies of the membrane and extracellular polysaccharide structures and physicochemical properties can make it possible to advance the understanding of both the role of these glycans in the adaptation of microorganisms to extreme conditions for existence and predict the possibility of their use for biotechnological purposes.

In this study, we determined the structure of the OPS from the moderately halophilic Gram-negative bacterium *Salinivibrio* sp. 9S8 isolated from Lake Qarun. The OPS has a linear regulate structure and is composed of tetrasaccharide repeating units. To our knowledge, this structure has not previously been found in bacterial polysaccharides [21]. A similar tetrasaccharide repeated unit decorated with pyruvate at 4,6 positions of Man was found in the OPS of *Raoultella terrigena* and *Shigella dysenteriae* Type 10 [29,30] as well as in the CPS from *Escherichia coli* O8:K50:H [31]. d-Man*p*4Lac is a rather rare component of bacterial polysaccharides. Until now, it has not been found in the OPS, but was reported as a component of the extracellular polysaccharides from *Cyanospira capsulate, Mycobacterium lacticolum*121 and *Mycobacterium album* B-88 [32,33,34].

Non-carbohydrate organic acids are often found in bacterial lipopolysachharides, for example, representatives of the *Proteus*, *Providencia*, *Shigella* [35,36]. It is believed that the presence of non-carbohydrate substituents and branching points in the polysaccharide chain increase the immunogenicity of the O antigen.

## 4. Materials and Methods

### 4.1. Sampling and Strain Isolation 

The strain *Salinivibrio* sp. EG9S8QL was isolated from a saline mud sample (salt mixed with clay collected from the bottom of solar salt ponds), collected in August 2018 from the Emisal salt company, Lake Qarun, Fayoum, Egypt (29°28′19.92″ N and 30°36′51.12″ E). Salt concentration of the tested sample was 18%. Strain EG9S8QL was isolated using S-G medium [37] supplemented with 1% glucose. The inoculated flask was incubated at 35 °C for 2 days at 200 rpm on a rotary shaker. Then, 0.1 mL of serial dilutions from the enriched sample was spread onto the surface of S-G agar plate medium and then incubated in sealed plastic bags at 35 °C for 2–3 days. Eleven isolates were isolated from the enriched sample. Strain EG9S8QL was selected for further phenotypic and genotypic characterizations. The growth factors (pH, NaCl content, temperature, carbon sources, cultivation duration) were studied to obtain the maximum bacterial growth as described [38]. The culture was stored at −80 °C in an S-G medium with final concentration 20% of glycerol.

### 4.2. Phenotypic and Genotypic Characterization of Strain EG9S8QL

The colony morphology of isolate EG9S8QL was described. Gram staining and the KOH string [39] tests were conducted. Bacterial motility and cell morphology were examined using phase-contrast microscopy with a DM2500 instrument (Leica, Germany). Hydrolysis of casein, gelatin and starch were determined as described [40]. Nitrate and nitrite reduction were tested as recommended [41]. Catalase activity was determined as described [42]. Other enzymatic activities were tested using API-20E (bioMérieux SA, Lyon, France) and BIOLOG GEN III MicroPlate^TM^ systems (BIOLOG^TM^, Cabot Blvd., Hayward, Berkeley Heights, NJ, USA), according to the instructions with 5% (*w*/*v*) NaCl. 

Strain EG9S8QL was identified by analyzing the sequence of the gene encoding 16S rRNA (16SrDNA). Genomic DNA extraction, amplification of 16S rRNA gene using universal primers fD1 (AGAGTTTGATCCTGGCTCAG) and rD1 (AAGGAGGTGATCCAGCC) [43], and purification of PCR product were conducted as described [38]. The nucleotide sequence of the purified PCR product was determined using an ABI 3730xL automated DNA sequencer (Applied Biosystems, Waltham, MA, USA) through Lab Biotechnology Company (Cairo, Egypt). The 16S rRNA gene sequences were initially analyzed using the program BLAST (National Center Biotechnology Information, http://www.ncbi.nml.nih.gov, 27 December 2018). The phylogenetic tree was designed as described [44] using *Escherichia coli* (NR114042.1) as an outgroup. Data of 16S rRNA gene sequence of strain EG9S8QL have been deposited in GenBank under accession number MZ646069.

### 4.3. Isolation of the Lipopolysaccharide and O-Specific Polysaccharide

The bacteria were cultivated in GRM medium supplemented with 5% NaCl to late exponential phase, and cells were harvested by centrifugation. The cells were repeatedly washed with 0.15 M NaCl, desiccated with acetone and air-dried. The biomass (8.8 g) was extracted by the Westphal procedure [21], proteins and nucleic acids were precipitated by CCl_3_CO_2_H (pH 2.7) and removed by centrifugation. After dialysis of the supernatant, an LPS preparation was obtained in 2.65% yield. The LPS was subjected to electrophoresis in 13% SDS polyacrylamide gel [45]. The components were visualized by staining the gels with a silver nitrate based dye [46].

LPS was O-deacylated under mild alkaline conditions (12.5% NH_4_OH, 37 °C, 16 h) and the resulting LPS-OH was isolated by exclusion chromatography on TSK HW-40 (S) (Tosoh Corporation, Tokyo, Japan) in 1% AcOH.

Fatty acid composition of the LPS and LPS-OH was determined after hydrolysis of the LPS with 1 M HCl in MeOH (80 °C, 16 h). The fatty acid methyl esters were analyzed at the Simbioz Center for the Collective Use of Research Equipment (IBPPM RAS) on a GC-2010 (Shimadzu, Kyoto, Japan) chromatograph equipped with an EQUITY-1 (30 m × 0.32 mm) (Sigma-Aldrich, St. Louis, MO, USA) column using the temperature program of 130 to 250 °C at 4 °C min^−1^; helium was used as a carrier gas at a flow rate of 1.3 mL min^−1^. The evaporator temperature was 260 °C and flow distribution was 1:50. Fatty acids were identified using the standard mixture of the fatty acid methyl esters (Sigma Aldrich, St. Louis, MI, USA).

An LPS sample (65 mg) was hydrolyzed with aq 2% AcOH at 100 °C for 3 h, a lipid precipitate was removed by centrifugation (13,000× *g,* 20 min), and the carbohydrate portion was fractionated by GPC on a column (56 × 2.6 cm) of Sephadex G-50 Superfine in 0.05 M pyridinium acetate buffer pH 4.5. The elution was monitored by a Knauer differential refractometer (Berlin, Germany). A high-molecular-mass OPS preparation was obtained in 29.2% yield.

### 4.4. Monosaccharide Analyses

The monosaccharides were analyzed as the alditol acetates after hydrolysis of the OPS with 2 M CF_3_CO_2_H (120 °C, 2 h) [47] and acetylated methyl glycosides after methanolysis of the OPS with acetylchloride in methanol (1:10, *v*/*v*, 100 °C, 4 h) by GC on an HP-5ms capillary column using an Agilent 7820A GC system and a temperature gradient of 160 °C (1 min) to 290 °C at 7 °C min^−1^. The absolute configurations of the monosaccharides were determined by GC of the acetylated glycosides with (*S*)-2-octanol, as described [48]. GC–MS was performed on a Hewlett Packard 5890 chromatograph (Palo Alto, CA, USA) equipped with a HP-5MS column and connected to a Hewlett Packard 5973 mass spectrometer (Palo Alto, CA, USA).

### 4.5. NMR Spectroscopy

^1^H and ^13^C NMR spectra were recorded on a Bruker Avance-III spectrometer (700.13 MHz for ^1^H and 176.04 MHz for ^13^C) using a 5 mm broadband inverse probehead for solutions in 99.95% D_2_O after deuterium exchange by freeze-drying sample solutions in 99.9% D_2_O. Acetone (δ_C_ 31.45, δ_H_ 2.225) was used as the internal calibration standard. The 2D NMR spectra were obtained using standard Bruker software, and Bruker TOPSPIN 2.1 program (Bruker Corporation, Billerica, MA, USA) was used to acquire and process the NMR data. The 2D TOCSY and ROESY spectra were recorded with a 180 ms duration of MLEV-17 spin-lock and a 200 ms mixing time, respectively. ^1^H,^13^C HMBC was optimized for an 8 Hz long-range constant.

## Figures and Tables

**Table 1:** ^1^H and ^13^C NMR data for the OPS from *Salinivibrio* sp. EG9S8QL (δ, ppm).

Residue	H-1 C-1 *^3^J*_1,2_	H-2 C-2 *^3^J*_2,3_	H-3 C-3 *^3^J*_3,4_	H-4 C-4 *^3^J*_4,5_	H-5 C-5 *^3^J*_5,6_	H-6(a, b) C-6
→2)-β-d-Man*p*4Lac-(1→, **A**	4.82 97.6 <2	3.88 77.1 ~4	3.80 74.5 ~10	3.60 78.3 ~10	3.45 77.3 ~6	3.76, 3.93 61.6
→3)-α-d-Man*p*NAc-(1→, **B**	5.05 96.5 <2	4.60 50.2 ~4	4.24 75.9 ~10	3.61 65.9 ~10	3.96 73.6 ~6	3.82, 3.84 61.3
→3)-β-l-Rha*p*-(1→, **C**	4.88 102.2 <2	4.29 67.8 ~4	3.67 77.8 ~10	3.46 71.3 ~6	3.46 73.4 ~6	1.33 18.0
→4)-α-d-Glc*p*NAc-(1→, **D**	5.07 99.6 3.7	3.87 55.2 ~10	3.99 71.4 ~10	3.74 78.6 ~10	4.17 71.2 ~10	3.69, 4.04 61.6
Lac, **L**	182.5	4.00 79.8	1.40 20.5			

## Supplementary Materials

The following are available online at https://www.mdpi.com/article/10.3390/md19090508/s1, Figure S1: Neighbour-joining tree showing the phylogenetic position of strain EG9S8QL (with blue color) and its related neighbour strains base d on 16S rRNA gene sequences. Bootstrap values (expressed as percentages of 100 replications) are shown at branch points. Bar 0.1 sub-stitutions per nucleotide position, Figure S2: Silver-stained SDS PAGE of the LPS from Salinivibrio sp. EG9S8QL (20 μg), Table S1: Comparative analysis of phenotypic features of strain EG9S8QL and closely related Salinivibrio species.
