# Supplementary material for: Structure of the 4-O-[1-Carboxyethyl]-d-Mannose-Containing O-Specific Polysaccharide of a Halophilic Bacterium Salinivibrio sp. EG9S8QL
Source: Mar Drugs. 2021 Sep 7;19(9):508. doi: 10.3390/md19090508 (PMC8466920; doi:10.3390/md19090508)
Supplement: Supplementary file 1 [file marinedrugs-19-00508-s001.zip › marinedrugs-1352052-supplementary.pdf]

Supplementary material
for

Structure of the 4-O-[1-Carboxyethyl]-D-Mannose-Containing O-specific Polysaccharide of a Halophilic Bacterium *Salinivibrio* sp. EG9S8QL

Elena N. Sigida,[1,2,*] Ibrahim M. Ibrahim,[3,4] Maxim S. Kokoulin,[5] Hussein H. Abulreesh,[6,7] Khaled Elbanna,[4,6,7] Svetlana A. Konnova,[1,3] and Yulia P. Fedonenko,[1,3]

[1] Institute of Biochemistry and Physiology of Plants and Microorganisms, Russian Academy of Sciences, 13 Prospekt Entuziastov, Saratov 410049, Russia; si_elena@mail.ru
[2] N. D. Zelinsky Institute of Organic Chemistry, Russian Academy of Sciences, 47 Leninsky Prospekt, Moscow 119991, Russia
[3] N. G. Chernyshevsky Saratov State University, 83 Ulitsa Astrakhanskaya, Saratov 410012, Russia
[4] Department of Agricultural Microbiology, Faculty of Agriculture, Fayoum University, Fayoum 63514, Egypt
[5] G. B. Elyakov Pacific Institute of Bioorganic Chemistry, Far Eastern Branch of Russian Academy of Sciences, 159 Prospekt 100 let Vladivostoku, Vladivostok 690022, Russia
[6] Department of Biology, Faculty of Applied Science, Umm Al-Qura University, Makkah, Kingdom of Saudi Arabia.
[7] Research Laboratories Unit, Faculty of Applied Science, Umm Al-Qura University, Makkah, Kingdom of Saudi Arabia
***** Correspondence: si_elena@ mail.ru; Tel.: (007-8452-970044)

Journal: Marine Drugs

The following are included as supplementary information for current paper:

Content

Table S1..……………………………………….…..............S2
Figure S1…………………………….…………………………………….………….…............S3
Figure S2..…………………………………………...…..…………….…............S4

Table S1. Comparative analysis of phenotypic features of strain EG9S8QL and closely related *Salinivibrio* species

Characteristics	Strains		
	EG9S8QL	*S. kushneri* LMG 29817^{Ta}	*S. costicola* DSM 11403^{Tb}
Site sampling	Lake Qarun, Egypt	water ponds of salterns, Spain	Hypersaline habitats
Cell morphology	Short, curved rods	Curved rods	Curved rods
Colony color	Cream-white	Cream	Cream
Nitrate reduction	-	+	-
NaCl range (optimum) (%, w/v)	3-20 (10)	2-20 (7.5)	0.5-20 (10)
Temperature range (optimum) (^{o}C)	10-43 (30)	17-49 (37)	5.0-45 (37)
pH range (optimum)	5.5-10 (8.0)	5.0-10 (7.4)	5.0-10 (7.5)
Utilization of			
Galactose	-	nd	-
Fructose	-	-	-
Mannose	+	+	-
Ribose	-	+	-
Xylose	+	-	+
Maltose	-	+	-
Lactose	-	nd	-
Trehalose	+	-	+
Glycerol	+	+	+
Na-acetate	+	nd	+
Hydrolysis of			
Starch	-	+	-
Tween 80	+	-	+

All strains are negative for spore formation, Gram reaction and utilization of arabinose and Na-citrate as a carbon source and positive for catalase and oxidase activities, gelatin and casein hydrolysis, and utilization of glucose and sucrose as a carbon source.
(+) growth or positive reaction, (−) no growth or negative reaction, (nd) no data
[a]data from [8], [b]data from Romano, I.; Gambacorta, A.; Lama, L.; Nicolaus, B.; Giordano, A. *Salinivibrio costicola* subsp. alcaliphilus subsp. nov., a haloalkaliphilic aerobe from Campania Region (Italy). *Syst Appl Microbiol*. **2005.** *28(1)*. 34-42; DOI: 10.1016/j.syapm.2004.10.001.

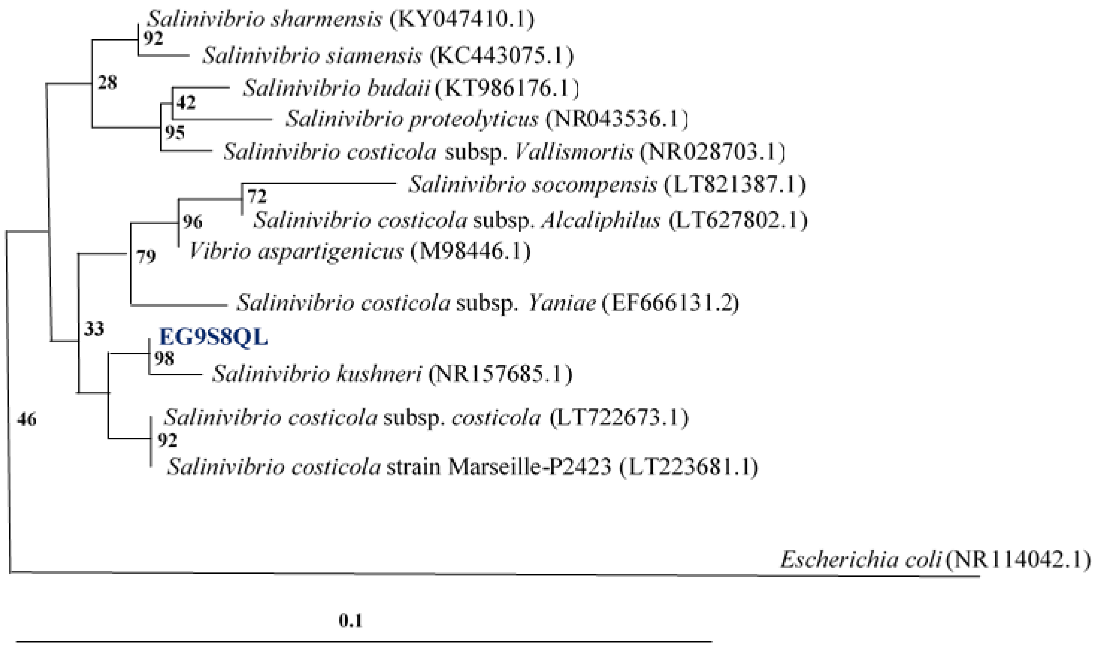

Figure S1. Neighbour-joining tree showing the phylogenetic position of strain EG9S8QL (with blue color) and its related neighbour strains base d on 16S rRNA gene sequences. Bootstrap values (expressed as percentages of 100 replications) are shown at branch points. Bar 0.1 substitutions per nucleotide position

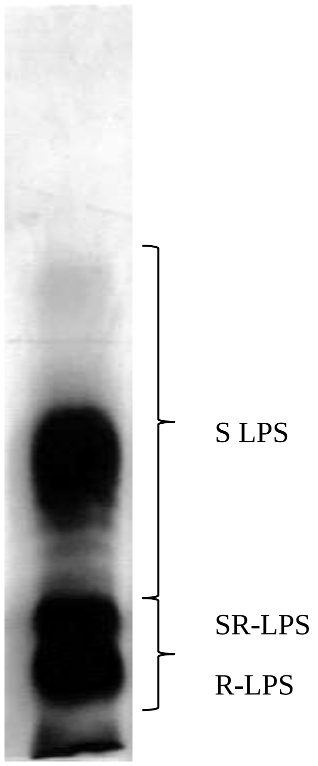

Figure S2. Silver-stained SDS PAGE of the LPS from *Salinivibrio* sp. EG9S8QL (20 μg)
